# Association of Gut Microbiota With Metabolism in Rainbow Trout Under Acute Heat Stress

**DOI:** 10.3389/fmicb.2022.846336

**Published:** 2022-03-30

**Authors:** Changqing Zhou, Shunwen Yang, Wei Ka, Pan Gao, Yalan Li, Ruijun Long, Jianlin Wang

**Affiliations:** ^1^State Key Laboratory of Grassland Agro-Ecosystems, Key Laboratory of Grassland Livestock Industry Innovation, Ministry of Agriculture and Rural Affairs, Grassland Agriculture Engineering Center, Ministry of Education, College of Pastoral Agriculture Science and Technology, Lanzhou University, Lanzhou, China; ^2^College of Ecology, Lanzhou University, Lanzhou, China; ^3^Gansu Fishery Research Institute, Lanzhou, China; ^4^Gansu Agriculture Technology College, Lanzhou, China

**Keywords:** rainbow trout, heat stress, gut microbiota, host metabolism, barrier function

## Abstract

Global warming is one of the most common environmental challenges faced by cold-water fish farming. Heat stress seriously affects the feeding, growth, immunity, and disease resistance of fish. These changes are closely related to the destruction of intestinal barrier function, the change of intestinal microbiota, and metabolic dysfunction. However, the causal relationship between the phenotypic effects of heat stress as well as intestinal and metabolic functions of fish is unknown. In the current study, the optimal growth temperature (16°C) of rainbow trout was used as the control group, while the fish treated at 22.5°C, 23.5°C, and 24.5°C for 24 h, respectively, were the treatment groups. The 16S rRNA gene sequencing analysis showed that with the increase in temperature, the relative abundance and diversity of intestinal microbiota decreased significantly, while the number of Mycoplasma, Firmicutes, and Tenericutes increased significantly. Non-targeted metabolomics analysis by liquid chromatography-mass spectrometry analysis and correlation analysis showed that the changes of metabolites related to amino acids, vitamins, and short-chain fatty acids in serum of rainbow trout under acute heat stress were strongly correlated with the decrease of relative abundance of various intestinal microbiota, especially *Morganella*, *Enterobacter*, *Lactobacillus*, *Lawsonia*, and *Cloacibacterium*. In addition, we also found that acute heat stress seriously affected the intestinal structure and barrier function, and also caused the pathological damage of epithelial cells. These results indicate that the gut microbiome of acute heat-stressed rainbow trout could mediate metabolite transfer through the gut barrier by affecting its integrity. Significant changes in gut morphology, permeability, antioxidant capacity, and pro-inflammatory cytokine levels were observed. Therefore, it is necessary to explore the changes of intestinal microbiota under heat stress to help understand the regulatory mechanism of heat stress and protect the intestinal health of rainbow trout from the negative effects of rising water temperature.

## Introduction

Stress refers to a series of systemic reactions caused by the destruction or interference of body homeostasis (Wendelaar, 1997; Zhou et al., 2019, 2021). With global climate change, heat stress has become a threat to fish farming. The rainbow trout (*Oncorhynchus mykiss*) is an artificially cultivated cold-water fish that is very sensitive to changes in water temperature. When the water temperature exceeds 18°C, the rainbow trout (adult fish) has a stress response, which may lead to reduced food intake, abnormal behavior, growth inhibition, and even death (Wendelaar, 1997; Huyben et al., 2019; Zhou et al., 2019). The gut is particularly sensitive to any type of stress, including heat stress (Sundh et al., 2010). A well-regulated intestinal function is critical for fish survival and production due to its significant impact on overall health and performance. Exposure to stress results in alterations of the brain-gut axis of animals (such as humans, cows, chickens, and pigs) ultimately leading to a series of inflammatory reactions and intestinal diseases (Konturek et al., 2011; Pearce et al., 2013; Koch et al., 2019; Pennisi, 2020; Cao et al., 2021). Although we are only beginning to understand how stress alters intestinal function by affecting the brain-gut axis, there is clear evidence in some species (such as cows, pigs, and chickens) that stress can lead to gastrointestinal dysfunction, as well as intestinal inflammation and infection (Pearce et al., 2013; Koch et al., 2019; Rostagno, 2020). Many studies have found that the bidirectional network regulation relationship between the brain and the digestive system is termed brain-gut axis (Rostagno, 2020). In other words, signals from the brain can change the sensory, secretory, and motor functions of the gut, whereas the information from the gut can in turn affect brain functions (Rostagno, 2020; Cao et al., 2021). Exposure to high temperature causes the activation of the hypothalamic-pituitary axis (HPA). For example, a rapid increase in the plasma corticosterone concentration and a decrease in the food intake, affects the integrity of intestinal epithelial cells (Lambert, 2009). Moreover, the activation of the autonomic nervous system (ANS) under heat stress increases the level of pro-inflammatory cytokines and increases intestinal permeability, which also affects the homeostasis of the intestinal environment (Ulrich-Lai and Herman, 2009; Chelakkot et al., 2018). Several studies have demonstrated that when the tight junction proteins (TJPs) between adjacent intestinal epithelial cells are destroyed, the permeability of the intestine increases, eases the penetration of harmful substances, thereby causing systemic reactions (Huyben et al., 2019; Koch et al., 2019). This is commonly referred to as “leaky gut” (Chelakkot et al., 2018).

The combination of microbiome and metabolome studies has been considered the most promising method for evaluating host-microbiome interactions (Turnbaugh and Gordon, 2008). Under extreme environmental conditions, the metabolic activities of animal gut microbiota are essential in maintaining host homeostasis and health, and changes in its composition can cause metabolic changes that lead to changes in the host phenotype (Visconti et al., 2019). A growing number of studies have shown that increased ambient temperature reduces the relative abundance and diversity of the gut microbiota of animals, which may have more negative effects on hosts, such as the Chinese giant salamander (Zhu et al., 2021), red-backed salamander (Fontaine et al., 2018), the common lizard (Bestion et al., 2017), and laying hens (Zhu et al., 2019). Huyben et al. (2018) reported that increased water temperature led to a decrease in the relative abundance of lactic acid bacteria in the intestinal contents of rainbow trout, whereas increases the relative abundance of Mycoplasmatales, which may have negative effects on intestinal health of fish. In addition, under different environmental conditions, intestinal microbiota can also change some phenotypes and characteristics of animals, which can accelerate the adaptability of animals to some extent (Alberdi et al., 2016; Macke et al., 2017; Romano, 2017). It has also been found that a fluctuating stress environment can also change the composition and diversity of intestinal microbiota that play a role in the host phenotypic variation (Sepulveda and Moeller, 2020).

Here, we hypothesized that the changes of intestinal morphology and tissue structure in rainbow trout under acute heat stress were related to the changes of intestinal microbiota. The gut microbial alterations changed the intestinal permeability and epithelial cell barrier structure, thus affecting the metabolic function of the host. Therefore, we used 16S rRNA gene sequencing combined with non-targeted metabolomics analysis (by liquid chromatography-mass spectrometry analysis) to investigate the effects of acute heat stress on the intestinal microbiota and host systemic metabolic activity of rainbow trout.

## Materials and Methods

### Fish and Facilities

The study was carried out in the Water Circulation Aquaculture System of Linxia Salmon-Trout Breeding Center (35°60′N 103°21′E; Linxia, China). The fish specimens were divided into 4 groups (1 control group and 3 treatment groups), with each group having 90 fish divided equally between 3 tanks. Each tank had 20 fish, and thus a total of 240 fish were allocated to 12 tanks. Each 600 L cylindrical cement tanks (diameter 100 cm) were equipped with 3 sets of heating (per 3000 W) and automatic temperature control equipment, which can raise the water temperature from 16°C to target temperature in 2 h. Rainbow trout were purchased from Linxia Salmon-Trout Breeding Center (Linxia, China), with the same generation background, similar body length and size, and with an average body weight of 182.4 ± 5.1g (mean ± SD). Before the exposure experiment, the fish were domesticated for two weeks under natural conditions, then the water temperature was controlled at 16°C for one week before the formal experiment began. During the experiment, three oxygen pumps (per 45 watts) were used to continuously supply oxygen and increase the dissolved oxygen in water, up to a concentration of 7 mg/L. Feed. The pellet feed was purchased from Beijing Han-Ye Technology Co., Ltd (Beijing, China; Crude Protein ≥ 48.0%, Crude Fat ≥ 10.0%) and the specimens were fed twice a day (at 8:00 a.m. and 17:00 p.m). The water source was an underground spring with a flow rate of 4–8 L/min, ammonia nitrogen content less than 0.2 mg/L, and pH of 7.4 ± 0.2. The study was conducted under natural light and in accordance with the requirements of the Animal Ethics Committee and was reviewed and approved by the Ethics Committee of School of Life Sciences, Lanzhou University (Approval number: EAF2021042).

### Sample Collection

According to our previous study (Zhou et al., 2019, 2021), the water temperature was rapidly increased from 16 to 22.5°C within 2 h, then this temperature was maintained (22.5°C) for 48 h, which led to ∼ 50% death of rainbow trouts (*n* = 90). Therefore, we speculated that 22.5°C may be a threshold for rainbow trout to cope with acute heat stress. In this study, a control group and three heat stress groups were incorporated. The optimal growth temperature for the control group was 16°C, while three (stress) treatment groups were maintained at 22.5°C, 23.5°C, and 24.5°C, respectively. Then, an acute heat stress model was established by continuous stress at each treatment temperature for 24 h. After the experiment, the fish were quickly anesthetized with 200 mg/L tricaine methanesulfonate (MS-222; Argent Chemical Laboratories, Redmond, WA, United States). Blood samples were collected from the caudal vein using a vacuum blood collection tube containing ethylenediamine tetraacetic acid (EDTA) anticoagulant and sterilized blood collection needle. The serum was obtained by centrifugation at 1000 rpm/min for 10 min. Later, each fish was dissected from the ventral side using sterile instruments on the ultra-clean workbench. The distal intestine (0.5 cm from the anus) of three fish from each tank was removed and collected as one sample for each of the groups and the intestinal contents were squeezed into the sterile Eppendorf tube with forceps. Finally, the mid-gut was separated and rinsed in PBS, one part was fixed with 4% paraformaldehyde, and the other part was placed in Eppendorf tubes at −80°C for preservation.

### Histology

The mid-gut of rainbow trout was fixed with 4% paraformaldehyde, dehydrated with ethanol, equilibrated in xylene, and embedded in paraffin. Tissue sections were cut (longitudinal cuts of ∼ 5 μm) using a tissue slicer (Leica, CM3050S), stained with hematoxylin and eosin (H.E), examined under a light microscope, and analyzed with ImageJ 1.5v image analysis software.

### Quantitative Real-Time Polymerase Chain Reaction Analysis

The midgut sample weighing 200 mg was taken into a mortar, and an appropriate amount of liquid nitrogen was added and the samples were grounded immediately to form a powder, then transferred to a sterile tube for total RNA extraction. Total RNA was extracted using TransZol™ Up Plus RNA Kit (TransGen Biotech, Beijing, China) according to the manufacturer instructions. The concentration and purity of total RNA were determined using a micro-spectrophotometer (ND-1000UV NanoDrop Technologies, Thermo Fisher, United States). 1 μL of the total RNA is reversely transcribed into 20 μL cDNA using a QuantiTect ^®^ Reverse Transcription Kit (QIAGEN, Germany) as per the standard kit protocol. To prevent sample contamination, the procedure was conducted in the laminar airflow hood. The reaction system was as follows: Total RNA 1.0 μl, gDNA Eraser 1.0 μl, 5 × gDNA Eraser Buffer 2.0 μl, RNase Free water 6.0 μl. The cycling parameters were as follows: 42°C for 15 min, and 85°C for 5 s. Relative quantitative expression was performed using a QuantiFast SYBR ^®^ Green real-time-polymerase chain reaction analysis (RT-PCR) Kit (QIAGEN, Germany) according to the manufacturer’s instructions. The primers used were synthesized by Sangon Biotech (Shanghai, China) Co., Ltd., and the primers for QRT-PCR are listed (Supplementary Table 1). Three technical replicates were employed per primer on a real-time quantitative PCR instrument (Thermo Fisher, ABI-7500, United States). The fluorescent dye was SYBR Green I, and the reference gene was β*-actin*. Reaction system (20 μl): 2 × QuantiFast SYBR Green RT-PCR Master Mix 10.0 μl; RNase-free water 6.2 μl; Template RNA 1.0 μl; Forward Prime 1.4 μl; Reverse Prime 1.4 μl. Reaction process: Pre-denaturation at 95°C for 15 min; Denaturation at 95°C for 10 s; Annealing at 57.6°C for 45 s; 95°C extension 15 s; 60°C, 1 min; 95°C extension 15 s; 40 cycles; 4°C, stored. Data were analyzed using QuantStudio™ Design and Analysis Software (Thermo Fisher, United States), and relative expression was calculated using the 2^–△△CT^ method.

### Gut Inflammation, Gut Permeability, and Stress Makers

Blood samples were collected from the caudal vein and centrifuged at 1000 rpm/min for 10 min to separate the serum. The Enzyme-Linked Immunosorbent Assay (ELISA) Kit was provided by Meimian Industrial Co., Ltd. (Jiangsu, China). Gut contents were analyzed for superoxide dismutase (SOD) activity, malonaldehyde (MDA), and glutathione (GSH) content. Serum was used to analyze diamine oxidase (DAO), lipopolysaccharide (LPS), interleukin 1β (IL-1β), interleukin 6 (IL-6), and TNF-α content.

### DNA Extraction and 16S rRNA Gene Amplification

The intestinal contents of 210 mg (per sample) were transferred to sterile tubes containing 0.5 g of 0.1 mm silica beads and homogenized in a Precellys homogenizer (Bertin Instruments, Montigny-le-Bretonneux, France) for two cycles of 60 s at 6000 rpm/min followed by 5 min rest on ice. DNA was extracted from the intestinal contents using a QIAamp Fast DNA Stool Mini Kit (Qiagen Gmbh, Hilden, Germany) according to product instructions. The concentration and purity of DNA were determined using Nanodrop 2000 (Thermo Fisher Scientific, Waltham, MA). DNA integrity was determined by 1% agar-gel electrophoresis. The V3-V4 regions of 16S rRNA were amplified by PCR using the forward primer 338F (5′-ACTCCTACGGGAGGCAGCA-3′) and the reverse primer 806R (5′-GGACTACHVGGGTWTCTAAT-3′). PCR conditions included denaturation at 95°C for 5 min, followed by 30 cycles of denaturation at 95°C for 30 s, hybridization at 50°C for 30 s, elongation 72°C for 60 s, and final elongation at 72°C for 5 min. Gel electrophoresis was used to confirm presence of the PCR products and the amplicons were purified, quantified using an enzyme mix (exonuclease I and Fast AP, Invitrogen) to eliminate free primers and dNTPs, according to Jaramillo and Castañeda (2021). The amplification conditions for the second PCR were set up as follows: denaturation at 94°C for 3 min, 8 amplification cycles at 95°C for 30 s, 55°C for 30 s, 72°C for 30 s, and a final extension at 72°C for 5 min. The PCR products were cleaned using the AMPure XT Bead kit (Beckman Coulter, Brea, CA, United States) and quantified using the Qubit Fluorometer and Qubit dsDNA HS assay kit. All libraries were diluted at 4 nM. Libraries were sequenced using the MiSeq Illumina platform at Baimaike Biotechnology Co., Ltd (Beijing, China) with a MiSeq Reagent kit v2 of 500 cycles.

### Quality-Filtering and Sequence Analysis

The library establishment and sequencing work was completed on the Illumina HiSeq 2500 platform (Beijing Baimaike Biotechnology Co., Ltd.). Data analysis was performed using BMKCloud.^1^ Data preprocessing included quality filtering, double-ended sequence splicing, and removal of chimera. The raw reads obtained by sequencing were filtered using Trimmomatic (version 0.33) software (Bolger et al., 2014). Then, Cutadapt (version 1.9.1) software was used to identify and remove the primer sequence, and clean reads without primer sequence were obtained (Kechin et al., 2017). The dual-end reads were spliced by Usearch (version 10) software, and the chimera was removed by Uchime (version 8.1) software to obtain effective reads (Edgar et al., 2011; Edgar, 2013). Information analysis included operational taxonomic units (OTUs) division, diversity analysis, difference analysis, and correlation analysis. The reads with 97.0% similarity were clustered using Usearch (Version 10.0) software, and the threshold was set to 0.005% for filtering to obtain OTU (Edgar, 2013). Using Silva (Release132^2^) as the reference database (Quast et al., 2013), naive Bayes classifier was used to make a taxonomic annotation for the feature sequences, and then the species classification information corresponding to each feature was obtained, and later the community composition of each sample was counted at all levels (phylum, class, order, family, genus, species).

QIIME2^3^ was used for bioinformatics analysis of the obtained data. Wilcoxon rank-sum test was used to analyze the Alpha diversity of intestinal microorganisms. Chao index was used to measure the relative abundance of bacteria, while Shannon and Simpson indexes were used to measure the diversity of bacteria. The Bray-Curtis algorithm was used to evaluate the Beta diversity of all samples, including principal component analysis (PCA) based on out, unweighted Unifrac Distance-based Principal Coordinate analysis (PCoA), and Binary-Jaccard-based non-metric multidimensional scaling (NMDS) analysis. Analysis of similarity (ANOSIM) test was used to assess the significant differences among groups. When the R value of ANOSIM was closer to 1, the difference among groups was greater than that within groups, and the closer the R value is to −1, the greater the intra-group difference as compared to the inter-group difference. Linear effect size feature selection (LEfSe) analysis^4^ was used to evaluate the significant differences among groups, using species with significant differences as biomarkers. Wilcoxon rank-sum test was used for difference analysis, and linear discriminant analysis (LDA) was used to estimate the effect of degree of these bacteria (from phylum to genus level). We identified the bacteria with the LDA score of “ ≥ 4” as “discriminative bacteria”.

### Association Analysis

The metabolites in rainbow trout serum were determined through high performance liquid chromatography coupled with triple quadrupole tandem mass spectrometry (LC-MS/MS). Data analysis was performed using BMKCloud (see text footnote 1). Before correlation analysis, all microbial OTUs were standardized, that is, the expression amount of each OTU divided by the total expression amount of all OTUs in the sample. In the calculation of correlation, metabolism and OTU were first processed by standardization of maximum and minimum values, and then the Pearson method was used to conduct correlation analysis of differential metabolites and OTU, according to the filter condition (|CC| > 0.80 and CCP < 0.05) with the result of correlation analysis. Finally, Cytoscape^5^ software was used to construct the interaction network of the selected microbe-metabolite pairs.

### Data Statistics and Analysis

All data were presented as the mean ± standard deviation (SD). *N* = 3 biological replicates (average of triplicate technical replicate per biological replicate) were employed. Statistical analysis of the difference test was performed by one-way analysis of variance (ANOVA) using “SPSS 22.0” and “Graphpad Prism7” software. “ns” indicated no significant difference; *P* < 0.05 was considered as a significant difference; *P* < 0.01 was moderately significant; *P* < 0.001 was extremely significant. *, *P* < 0.05; **, *P* < 0.01; ***, *P* < 0.001 using a one-way analysis of variance (ANOVA) followed by Tukey’s HSD test.

## Results

### Intestinal Morphology

**FIGURE 1:**
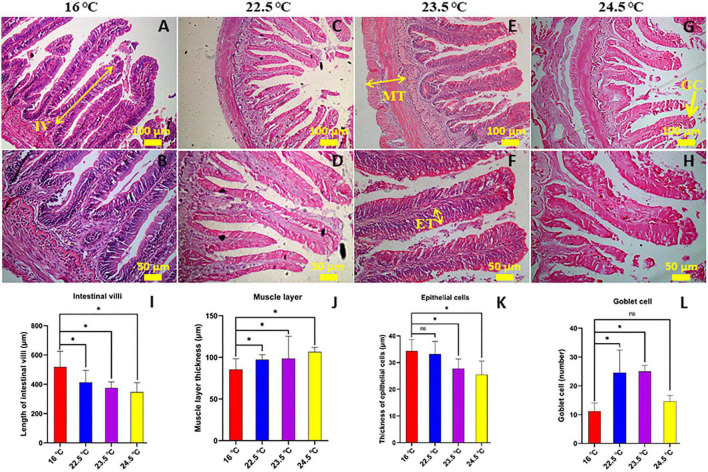
Effects of acute heat stress on the intestinal morphology [the mid-gut stained with H-E]. **(A,B)** 16°C control group; **(C,D)** 22.5°C-stress group; **(E,F)** 23.5°C-stress group; **(G,H)** 24.5°C-stress group. IV, length of intestinal villi (*N* = 15); MT, muscular layer thickness (*N* = 10); ET, thickness of epithelium cell (*N* = 25); GC, goblet cell number. Scale bars **(A,C,E,G)** = 100 μm; **(B,D,F,H)** = 50 μm. **(I–L)** the measured of mid-gut IV, MT, ET, and GC, respectively. *P < 0.05; ns, no significance.

Histological study showed that the intestinal histology was significantly affected by temperature change, and obvious pathological features appeared in the gut. In the present study, morphological and pathological changes in the mid-gut of rainbow trout were analyzed in depth (Figure 1). At the histological level, with the increase of stress temperature, intestinal villus height and epithelial cell thickness decreased gradually, but muscle thickness and goblet cell number increased significantly. In addition, intestinal tissue injury was serious in 24.5°C-stress group, including fracture of some intestinal villi and fissure of the muscle layer. These results indicate that the intestinal morphology and structure of rainbow trout have undergone significant changes in order to adapt to the high temperature environment, and there was serious pathological damage in the gut.

Inflammatory levels in intestinal tissues were assessed using a semi-quantitative scoring system (Hu et al., 2016). The results showed that compared with the control group, the intestinal lamina propria in 22.5°C-stress group had mild inflammatory infiltration, slightly enlarged lamina propria, increased goblet cell number, and relatively intact intestinal villi and epithelial cells. In the 23.5°C-stress and 24.5°C-stress groups, intestinal tissue showed an obvious inflammatory response (Table 1). Due to the infiltration of inflammatory cells, the width of lamina proper and submucosa was significantly increased, and the connective tissue and the number of goblet cells between the bottom of the folds and the dense layer were significantly increased. These results indicate that with the increase of heat stress intensity, the degree of intestinal inflammatory response and tissue damage of fish is more serious.

**TABLE 1:** Pathological injury scores of rainbow trout mid gut under acute heat stress.

	16°C		22.5°C		23.5°C		24.5°C	
	Mean	SEM	Mean	SEM	Mean	SEM	Mean	SEM
Goblet cells	11.20^b^	1.28	24.60^a^	3.48	25.00^a^	0.95	14.60^b^	0.92
Lamina propria inflammation	1.20^b^	0.20	2.80^a^	0.20	3.20^a^	0.20	3.60^a^	0.40
Villus damage	1.20^d^	0.20	2.20^c^	0.20	3.40^b^	0.24	4.40^a^	0.24
Edema	1.20^c^	0.20	2.40^b^	0.24	3.20^b^	0.20	4.20^a^	0.20

*A score of 1-2 represents normal morphology, a score of 3–4 was given to distinct morphological signs of inflammation, while a score of 5 represents severe symptoms of enteritis. Means ± SEM; different letters (a, b, c) indicate a statistically significant difference among groups at significance level p < 0.05.*

### Intestinal Barrier

**FIGURE 2:**
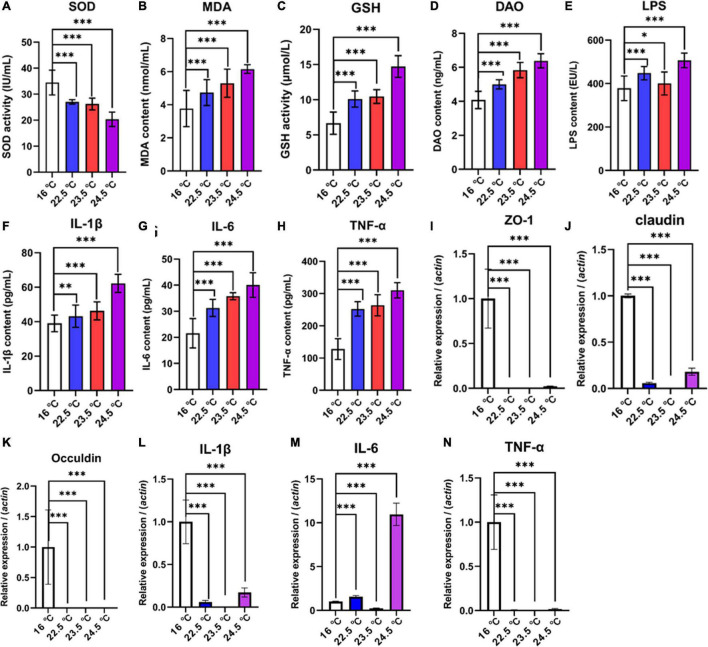
Effects of acute heat exposure on intestinal barrier and antioxidant capacity. **(A–C)** the antioxidant capacity of intestinal contents was detected by enzyme linked immunosorbent assay (ELISA), which were SOD, MDA, and GSH, respectively. **(D,E)** DAO and LPS were used to detect intestinal permeability in serum by ELISA. **(F–H)** the levels of pro-inflammatory cytokines in serum were detected by ELISA, including IL-1β, IL-6, and TNF-α, respectively. **(I–K)** expression of tight junction protein-related genes in mid gut were determined by quantitative real-time PCR, which were Zonula Occludens 1 (*ZO-1*), *Claudin*, and *Occludin*, respectively. **(L–N)** expression of pro-inflammatory cytokines related genes in mid gut were determined by quantitative real-time PCR, which were IL-1β, IL-6, and TNF-α, respectively. The reference gene is β*-actin.* Bars represent geometric means ± standard deviation. *N* = 3 biological replicates (average of triplicate technical replicate per biological replicate). *, *P* < 0.05; **, *P* < 0.01; ***, *P* < 0.001 using a one-way analysis of variance (ANOVA) followed by Tukey’s HSD test.

Intestinal permeability, antioxidant capacity, and pro-inflammatory cytokine levels of rainbow trout were significantly changed under acute heat stress (Figures 2A–H). Compared with the control group, SOD activity of antioxidant factor in intestinal contents decreased significantly with the increase of stress intensity, while MDA and GSH contents increased significantly. The results indicated that the physiological load of rainbow trout was increasing and the antioxidant capacity was gradually decreasing under the acute heat stress. Compared with the control group, DAO and LPS levels in serum were significantly increased, representing that intestinal permeability of rainbow trout increased under acute heat stress. Compared with the control group, the serum levels of pro-inflammatory factors IL-1β, IL-6, and TNF-α were drastically increased, suggesting that there may be an inflammatory response in the intestine of rainbow trout under acute heat stress, and the change may be related to the oxidative stress.

To investigate the association between increased intestinal permeability and TJPs changes under acute heat stress, a relatively quantitative analysis of selected genes encoding TJPs in the mid-gut of rainbow trout was performed. QRT-PCR data revealed that compared with the control group, the mRNA relative abundance of Zonula Occludens 1 (*ZO-1*), *Claudin 1*, and *Occludin* in all treatment groups was significantly reduced (Figures 2I–N). In addition, the gene expression of selected specific markers for intestinal stress, inflammation, and immune defense in the gut mucosa of rainbow trout were altered. The results showed that the mRNA relative abundance of IL-1β, IL-6, and TNF-α decreased in 22.5°C-stress, 23.5°C-stress, and 24.5°C-stress groups. There was no significant difference in IL-6 mRNA relative abundance between the 22.5 and 23.5°C-stress groups, but significantly increased in 24.5°C-stress group. These results suggest that as the intensity of acute heat stress increases the degree of intestinal permeability and pathological damage, ultimately disrupting intestinal homeostasis.

### Intestinal Microbial Analysis

**FIGURE 3:**
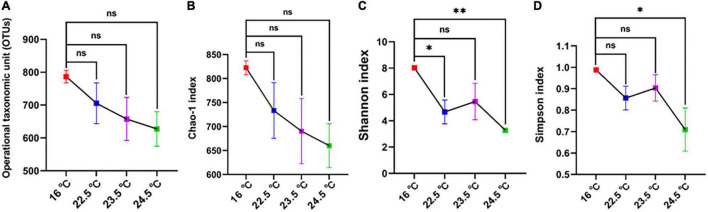
Comparison of OTU levels and alpha diversity of intestinal microbiome in different temperature groups. **(A)** OTUs; **(B)** Chao-1 index; **(C)** Shannon index; **(D)** Simpson index; *P < 0.05; **P < 0.01; ns, no significance.

The diversity and relative abundance of rainbow trout intestinal microbiota were significantly affected by acute heat stress. The processed sequencing data gave a total of 956,089 clean reads obtained from twelve samples. The quality of sequencing data of each sample was further statistically evaluated (Supplementary Table 2). Reads were clustered at a similarity level of 97%, and the number of OTUs in each sample ranged from 529 to 819 (Figure 3A).

The alpha diversity of intestinal microbiota showed that Chao-1, Shannon, and Simpson indices decreased with the increase of stress temperature (Figures 3B–D). Compared with the control group, the Chao-1 index of all treatment groups decreased gradually, and there was no significant difference between the groups. The Shannon index of the 22.5°C-stress group and 24.5°C-stress group decreased significantly, but the decrease of the 23.5°C-stress group was not significant. There was no significant decrease in the Simpson index of the 22.5°C-stress and the 23.5°C-stress groups, while the decrease in the 24.5°C-stress group was significant.

**FIGURE 4:**
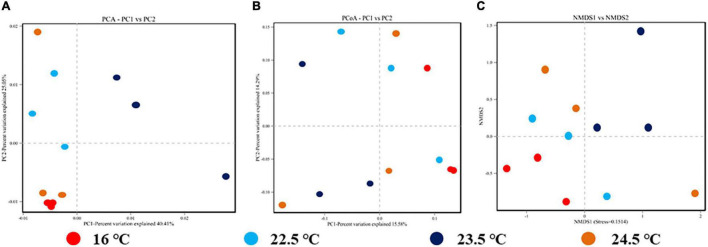
Beta diversity analysis of intestinal microbiome for each sample in different temperature stress groups. **(A)** OTUs-based PCA plot; **(B)** Unweighted Unifrac distance-based PCoA plot; **(C)** Binary-Jaccard distance-based NMDS plot.

Through beta diversity analysis, different samples were separated significantly on PCA, PCoA, and NMDS two-dimensional coordinate plots, indicating significant differences in species diversity between samples and reliable data (Figure 4). The composition and distribution of the intestinal microbiome in four groups could be visually separated, and the ANOSIM test confirmed the significance (PCA, PCoA, and NMDS: *P* < 0.01). The results showed that the similarity of intestinal microbiota composition of rainbow trout decreased with the increase of stress temperature. In brief, the decrease of intestinal microbiota relative abundance and diversity during acute heat exposure is positively correlated with the decrease of adaptation ability to the high temperature environment.

With the increase of stress temperature, the number and composition of intestinal microbiota changed markedly. At the phylum level, Proteobacteria, Firmicutes, Tenericutes, Fusobacteria, and Bacteroidetes were the most influenced phyla in the gut (Supplementary Figure 1). At the genus level, acute heat stress significantly increased the proportion of *Mycoplasma*, *Cetobacterium*, *Aeromonas*, *Shewanella*, and *Clostridium* (Supplementary Figure 2). The proportion of rainbow trout intestinal microbiota at phylum and genus level is shown in Supplementary Tables 3, 4. The unweighted pair-group method with an arithmetic mean (UPGMA) method was used to conduct clustering and species similarity analysis for different samples. The results showed that the species similarity of the samples within the group was high, while the species composition distance between the groups was far (Supplementary Figure 3). Compared with the control group, *Mycoplasma*, *Cetobacterium*, *Aeromonas*, *Shewanella*, *Clostridium*, *Bacteroides*, and *Enterobacter* accounted for the largest proportion of intestinal microbiota in the heat stress group. These bacteria can become the main gut microbiota of rainbow trout under heat stress in the future.

The similarity analysis of intestinal microbiome in different stress temperature groups showed that the samples were significantly different between groups, and the test reliability was high (Supplementary Figure 4) (R2 = 0.618, Pvalue = 0.001). LEfSe analysis identified nineteen discriminative bacteria (from phylum to genus level) among the four groups (Supplementary Figure 5). *Cloacibacterium*, Prevotellaceae, *Microbacterium*, *Morganella*, and Lactobacillus-fermentum were more abundant in the control group. *Mycoplasma*, Mycoplasmataceae, Mycoplasmatales, Tenericutes, and Mollicutes were more abundant in the 22.5°C-stress group. Firmicutes was more abundant in the 23.5°C-stress group, whereas Betaproteobacteriales and Bacteria were more abundant in the 24.5°C-stress group.

**FIGURE 5:**
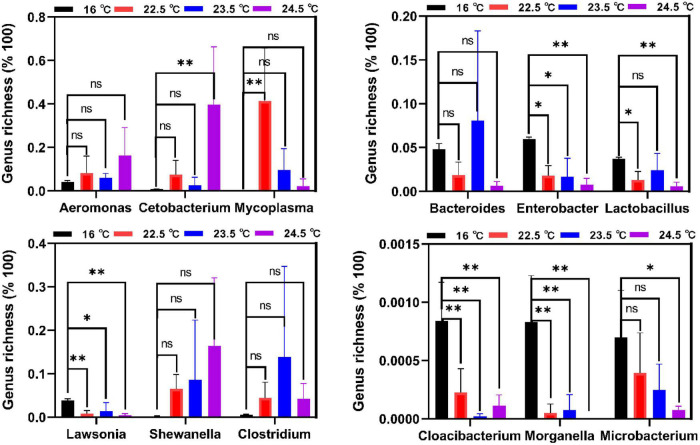
Biomarkers of discriminative bacteria (genus) in different temperature groups identified. *P < 0.05; **P < 0.01; ns, no significance.

According to the differential analysis of intestinal microbiota composition, Tenericutes and Firmicutes are biomarkers at the phylum level under acute heat stress (Supplementary Figure 6), and the differences were more obvious at the genus level. In Figure 5, *Cloacibacterium*, *Microbacterium*, and *Morganella* were the dominant bacteria in the control group, while their relative abundance was reduced in all three heat stress groups. Compared with the control group, the relative abundance of *Cloacibacterium* and *Morganella* decreased significantly in the 22.5°C-stress, 23.5°C-stress, and 24.5°C-stress groups (*P* < 0.01). Compared with the control group, *Microbacterium* showed no significant decrease in the relative abundance of the 22.5°C-stress and 23.5°C-stress groups, but was significant in the 24.5°C-stress group (*P* < 0.05). Compared with the control group, the relative abundance of *Enterobacter*, *Lactobacillus*, and *Lawsonia* were significantly decreased in all three heat stress groups. In addition, *Aeromonas*, *Cetobacterium*, *Mycoplasma*, *Shewanella*, and *Clostridium* increased in all three heat stress groups, but there was no significant difference compared with the control group.

### Associations Between Microbiota and Metabolites

**FIGURE 6:**
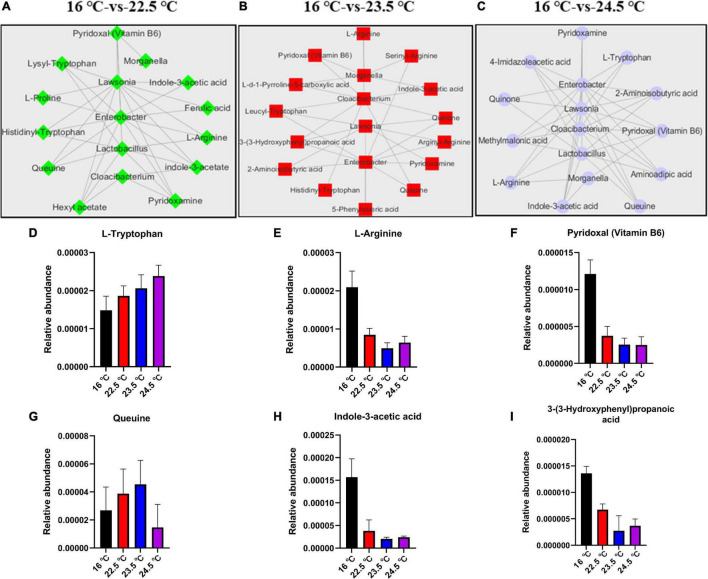
Associations of intestinal microbiome with serum metabolites in rainbow trout under acute heat stress. **(A–C)**. The correlation network analysis of 16°C-vs-22.5°C, 16°C-vs-23.5°C, and 16°C-vs-24.5°C, respectively. **(D–I)**. Histogram of relative abundance of some metabolites in serum.

To identify the correlation between intestinal microbes and serum metabolites in rainbow trout under acute heat stress, co-occurrence network using Pearson correlation coefficient (CC, |cc| > 0.80, *P*-_cc_ < 0.05) was conducted. There were 9,883 associations between 631 OTUs and 177 metabolites in the 16°C-vs-22.5°C group, 9,548 associations between 285 OTUs and 151 metabolites in the 16°C-vs-23.5°C group, 22,276 associations between 355 OTUs, and 177 metabolites in the 16°C-vs-24.5°C group. Here, we selected 5 intestinal microbiota (including *Morganella*, *Enterobacter*, *Lactobacillus*, *Lawsonia*, and *Cloacibacterium*) with the most significant differences between groups under acute heat stress for serum metabolite correlation analysis, and established an interaction network (Figures 6A–C). In Figure 6, there were 34 pairs in the 16°C-vs-22.5°C group, of which 22 were positively correlated and 12 were negatively correlated. Furthermore, there were 30 pairs in the 16°C-vs-23.5°C group, of which 12 were positively correlated and 18 were negatively correlated, while out of the 44 pairs in the 16°C-vs-24.5°C group, 14 were positively correlated and 30 were negatively correlated. This suggests that heat stress significantly alters the composition of intestinal microbiota in rainbow trout. These “heat microbiota” remodel the body’s metabolism of amino acids, vitamins, and short-chain fatty acids (SCFAs) (Figures 6D–I), reducing the host’s ability to adapt to high temperatures. Under acute heat stress, compared with the control group, the relative abundance of L-Tryptophan in serum gradually increased, but the relative abundance of L-arginine gradually decreased. The relative abundance of vitamin B6 in serum decreased under acute heat stress, while the relative abundance of queuine increased in 22.5°C and 23.5°C groups, but decreased in 24.5°C group. In addition, the relative abundance of indole-3-acetic acid and 3-(3-Hydroxyphenyl) propanoic acid in serum decreased under acute heat stress. These results indicate that there is a close relationship between gut microbiota and host metabolites in rainbow trout under acute heat stress.

## Discussion

The physiological regulation of heat stress is a complex scientific problem that has been studied in many animals, such as humans, rats, livestock, and fish (Wendelaar, 1997; Koch et al., 2019; Pennisi, 2020; Wei et al., 2021). Therefore, this study took rainbow trout as the research object to investigate the effects of acute heat stress on intestinal morphology and tissue structure, the changes in intestinal microbiota composition and abundance, and the effects on serum metabolites of the host, to analyze the physiological regulation mechanism of cold-water fish under high temperature environments.

### Gut Microbiota

Changes in the environmental temperature can reshape the intestinal microbiota of animals, and changes in intestinal microbiota can in turn affect the phenotype and adaptability of the host (Wernegreen, 2012; Alberdi et al., 2016). In the current study, alpha diversity (including Chao1, Shannon, and simpson index) analysis results showed that different temperature stress significantly reduced the diversity and richness of rainbow trout intestinal microbiome, and with the increase of stress intensity, the decrease of these indicators became more significant. Previous studies have shown that heat stress significantly reduces the diversity of gut microbiota in rainbow trout, broilers, and pigs (Song et al., 2014; Sohail et al., 2015; Huyben et al., 2018; Xiong et al., 2020). The inconsistent results in our study may be due to different species and different intensities of temperature stress. Bestion et al. (2017) found that climate warming (2–3°C) resulted in a 34% loss of gut microbiota diversity in the common lizard (*Zootoca vivipara*), with possible negative consequences for host survival. The species and quantity of intestinal dominant microbiota varied significantly under different environmental conditions. In the current study, compared with the control group, the numbers of *Lactobacillus*, *Enterobacter*, *Bacteroides*, and *Lawsonia* in the gut of rainbow trout under acute heat stress significantly decreased, while the numbers of *Mycoplasma*, *Cetobacterium*, *Aeromonas*, *Shewanella*, *Clostridium*, and *Romboutsia* significantly increased. Similar results were reported in Huyben et al. (2018; 2020) work, in which an increased water temperature significantly altered the gut microbiota of rainbow trout, leading to a decrease in the abundance of lactic acid bacteria (especially *Lactobacillus*) and an increase in the abundance of Mycoplasmatales. These results suggest that the changes in the number of these intestinal bacteria are closely related to the increase of temperature in the environment, and the damage of intestinal epithelial cells and intestinal barrier function in rainbow trout under heat stress are related to the changes in the number of these bacteria. Therefore, we believe that the changes in intestinal microbiota structure and relative abundance under acute heat stress may be more detrimental to the survival of rainbow trout.

Under different stress intensities, the number and proportion of intestinal microbiota in rainbow trout were also different. According to LEfSe analysis results, *Cloacibacterium*, *Microbacterium*, and *Morganella* dominated the intestinal microbiota of the control group. However, the intestinal microbiota of rainbow trout in acute heat stress accounted for the largest proportion of Tenericutes, Mycoplasma, and Firmicutes. Firmicutes are considered to be the main butyrate-producing bacteria (Parada et al., 2019). In the current study, the proportion of Firmicutes in the 24.5°C-stress group significantly decreased under acute heat stress (Supplementary Figure 6), suggesting that the destruction of intestinal mucosal barrier and disturbance of intestinal ecological environment under heat stress may be related to changes in Firmicutes and SCFAs abundance (Koh et al., 2016; Jin et al., 2018). At present, there are few studies on *Mycoplasma* in the salmon gut, and its functions and effects are not clear. Etyemez and Balcázar (2015) reported that *Mycoplasma* was the most abundant species in rainbow trout gut and was not specifically associated with any known species. Firmicutes or Mycoplasma were found to be the dominant microbiota (83%) in the gut of rainbow trout (Lyons et al., 2017; Huyben et al., 2018) and 92% in Atlantic salmon (Zarkasi et al., 2014; Llewellyn et al., 2016; Rudi et al., 2018). Mycoplasma can produce nutrients but is also associated with tumors in fish, so it is both beneficial and harmful. Huyben et al. (2021) found an increase in intestinal abundance of Tenericutes, especially *Mycoplasma* (genus) and Mycoplasmataceae (order), in Atlantic salmon fed the high lipid diet. These changes in gut microbes may be associated with increased activation of host energy and carbohydrate metabolic pathways. *Mycoplasma penetrans*, the most common species of *Mycoplasma*, has been found to be a candidate agent of transmissible tumors in the intestine of zebrafish (Burns et al., 2018). A large amount of *Mycoplasma* was also noted in fish during the transmission of intestinal tumors (Burns et al., 2018). In general, Firmicutes may be the signature intestinal bacteria for rainbow trout to cope with the adverse environment, but the cognition and reports of Mycoplasma and Tenericutes are relatively few. Their functions and effects in rainbow trout need to be further studied and verified.

According to current research results, the relative abundance and diversity of intestinal microbiota in rainbow trout are decreased under acute heat stress. These results further illustrate that the changes of the intestinal microbiota are complex, in which the effect of single microbiota may be relatively small, but the interaction between multiple microbiota is important.

### Gut Morphology

Previous studies have found that heat stress has little effect on the intestinal morphology and structure of cows and pigs, and the villi height of gut may not be specifically controlled by heat stress, but rather affected by reduced energy and nutrient intake (Pearce et al., 2013; Koch et al., 2019). Our results show that heat stress caused direct damage to the intestinal morphology and epithelial cells of rainbow trout. Huyben et al. (2019) found that water temperature rising from 11 to 18°C resulted in villous damage and edema in the proximal intestine of rainbow trout. There may be two reasons why the intestinal damage of cows, pigs, and chickens is relatively weak under heat stress: (1) due to different species; (2) is due to the relatively weak stress intensity and relatively short duration. In the current study, intestinal morphology and structure of rainbow trout were significantly changed under acute heat stress. With the increase of stress temperature, intestinal villus height and epithelial cell thickness decreased gradually, but muscle thickness and goblet cell number increased significantly. According to the semi-quantitative scoring criteria (Hu et al., 2016), the intestinal inflammatory response of rainbow trout under acute heat stress increases, and leads to serious tissue damage. In addition, after several consecutive tissue sections and H.E staining, we confirmed that intestinal villi in the 24.5°C-stress group were fractured and pathologically damaged. Therefore, our results indicate that acute heat stress has significant effects on intestinal morphology and histopathology of rainbow trout.

In extremely hot conditions, reduced blood flow to the gut causes intestinal leakage, which in turn causes inflammation (Pennisi, 2020). The intestinal epithelium is composed of a single layer of intestinal cells, which are connected by TJPs between adjacent enterocytes to form a protective barrier of selective infiltration, known as the intestinal barrier (Rostagno, 2020). This barrier allows absorption of nutrients (such as amino acids, electrolytes, and water) while avoiding infiltration of harmful substances such as pathogens. Huyben et al. (2019) found that a rise in water temperature from 11 to 18°C reduced the expression of inflammatory cytokines (TNF-α and IL-8) in the proximal intestinal of rainbow trout. In the current study, intestinal permeability, serum pro-inflammatory cytokines, and antioxidant capacity of rainbow trout were significantly increased under acute heat stress. These results indicate that the intestinal barrier function and the homeostasis of intestinal environment is disrupted under acute heat stress. Several biomarkers, including heat stress proteins, tight junctions, cytokines, oxidative stress factors, and their tissue expression levels, are frequently used to evaluate intestinal damage caused by heat stress (Varasteh et al., 2015). Sundh et al. (2010) found that the intestinal barrier function of Atlantic salmon living under adverse environmental conditions for a long time would be significantly reduced, and this is a good experimental marker for the evaluation of chronic stress in fish. Loss of the gut integrity, leads to LPS entry into the blood stream, resulting in innate immune system activation and systemic inflammation (Matthes and Gisolfi, 2001). It has been suggested that heat stress can directly alter TJPs in the jejunum of cows, causing damage to the intestinal barrier, and an uncategorized cell (myeloic origin and macrophage-like phenotype) is induced into the jejunum mucosa and submucosa, producing inflammatory cell infiltration (Farquhar and Palade, 1963; Hollander, 1988). Similarly, the increased intestinal permeability in poultry under heat stress is caused by disruption of intracellular and intercellular tight junctions (Song et al., 2014; Wu et al., 2018). In the current study, the relative expression levels of intestinal TJPs and pro-inflammatory cytokine related genes were significantly decreased in rainbow trout under acute heat stress, suggesting that acute heat stress directly damaged the intestinal barrier function of rainbow trout, thus breaking the intestinal homeostasis.

### Analysis of Interaction Between Gut Microbiota and Serum Metabolites

Previous studies on the gut microbiome have focused on individual microbial taxa, while the metabolic potential of microbes and the association between microbial metabolism and host metabolism have been largely ignored (Visconti et al., 2019). In this study, we investigated the effects of acute heat stress on the intestinal microbiota and host metabolic function of rainbow trout by analyzing the correlation between intestinal microbiota and serum metabolite data. Interestingly, among the numerous microbiome–metabolome associations identified in this study, a large proportion was negatively correlated, which was involved in the metabolism of amino acid, vitamin, and SCFAs.

In our study, acute heat stress strengthened the association between the characteristic gut microbiota of rainbow trout and host amino acid metabolism. Changes in the species and composition of intestinal microbiota can affect the host’s digestion and absorption of amino acids (Lin et al., 2017). For example, *Clostridium* is the key driver of amino acid fermentation, while *Peptostreptococcus* is the key driver of glutamate or tryptophan utilization (Dai et al., 2011). L-Tryptophan is one of the nine essential amino acids, whose metabolism appears as an important inflammatory inhibitor and modulator of gut microbiota, with significant effects on physiological and pathological pathways (Chen et al., 2018; Taleb, 2019). The level of L-Tryptophan was significantly increased in the three treatment groups, and the interaction network showed that it was negatively correlated with *Bacteroides*, *Enterobacter*, *Cloacibacterium*, *Lawsonia*, and *Psychrobacter*. These five bacteria play an important role in amino acid metabolism. Studies have shown that the characteristics and distribution of intestinal microbiota are key determinants of the level of Tryptophan metabolites in the systemic circulation (Lin et al., 2017). These results suggest that dietary L-Tryptophan supplementation may improve the intestinal function of rainbow trout to cope with stressful environments. It has been suggested that L-arginine supplementation can protect intestinal health by inhibiting local inflammatory response, promoting the production of tight junctions, and inducing autophagy to alleviate intestinal injury caused by heat stress (Huang et al., 2020). In the current study, decreased serum L-arginine level under acute heat stress was positively correlated with the characteristic intestinal microbiota, including *Lawsonia*, *Lactobacillus*, and *Enterobacter*. This indicated that L-arginine metabolism and absorption of host under heat stress are closely related to changes in intestinal microbiota relative abundance. L-arginine enhances the ability of *Caenorhabditis elegans* to resist heat stress by enhancing the scavenging ability of free radicals and up-regulating the expression of aging-associated genes (Ma et al., 2016). In addition, L-proline metabolism is significantly correlated with intestinal microbiota *Lawsonia* and *Enterobacter*, both of which are negatively correlated.

In our study, acute heat stress strengthened the association between characteristic gut microbiota of rainbow trout and host vitamin metabolism. Vitamins play an important role in maintaining normal physiology and regulating metabolic function in humans and animals (Ames, 2018) under diseases such as scurvy, beriberi, anemia, rickets (Ames, 2003). Extreme stress caused host metabolic dysfunction, and serum levels of vitamin-related metabolites, including Vitamin B and queuine, were significantly altered. Correlation analysis showed that host metabolism was driven by changes in intestinal microbiota under heat stress, and the bacteria with strong correlation included *Morganella*, *Enterobacter*, *Lactobacillus*, *Lawsonia*, and *Cloacibacterium*. The levels of vitamin B6 in rainbow trout serum decreased under heat stress. More than 700 microbes linked to vitamin B-related metabolites have been found in the human gut in recent studies (Visconti et al., 2019). Vitamin B in animals is mainly absorbed through diet, followed by the synthesis of lactic acid bacteria in the intestine (LeBlanc et al., 2013). In addition, we found that metabolic abnormalities of pyridoxamine and vitamin D2 are closely associated with the changes in intestinal microbiota. The decrease of animal feeding and the change of intestinal microbiota under heat stress affects metabolic functions and endanger health. Interestingly, in this study, we found that the numerous gut microbiota were associated with queuine (serum metabolite). Under acute heat stress, serum queuine levels in rainbow trout increased in 22.5°C-stress group and 23.5°C-stress group, but decreased in 24.5°C-stress group. Queuine is an evolutionarily ancient micronutrient (Nishimura, 1983). Although mainly supplied to the host through food (e.g., tomatoes, wheat, milk), it can also be synthesized by eubacteria (Fergus et al., 2015). In the heat stress environment, the feeding of rainbow trout decreased or even stopped (food intake was not measured in our study), the intestinal microbiota was disturbed, and the level of queuine decreased or was deficient, leading to the decrease of the cofactor tetrahydrobiopterin (BH4) (Rakovich et al., 2011). BH4 is a cofactors in biotransformation of various trace elements, such as Phenylalanine, Tryptophan, Tyrosine, and Arginine (Watschinger et al., 2015; Alrayes et al., 2016). It is thought that queuine-deficient mice develop tyrosine deficiency and die within 18 days of their withdrawal (Marks and Farkas, 1997). It can be concluded that there is a significant correlation between intestinal microbiota and host metabolism under acute heat stress, and this correlation may be even stronger under chronic stress.

In our study, acute heat stress strengthened the association between the characteristic gut microbiota of rainbow trout and host SCFAs metabolism. SCFAs metabolism has been associated with gut microbiota composition in many studies (Cui et al., 2019; Parada et al., 2019; Zhang et al., 2021). Under acute heat stress, the metabolism of fatty acids in serum of the host, especially SCFAs, was significantly changed, and mainly indole-3-acetic acid and 3-(3-Hydroxyphenyl) propanoic acid were down-regulated, while 2-aminoisobutyric acid and valeric acid were up-regulated. Relevant studies suggest that SCFAs (mainly acetic, butyric, and propionic acids) are the most abundant metabolites produced by microbial fermentation of carbohydrates in the gut, and are important in the physiological activities of microbiota and host (Sakata, 1987; Zhang et al., 2021). Butyrate has an important immunomodulatory function that is the main product of intestinal microbial fermentation and plays an important role in regulating the functional activities of host metabolism, including thermogenesis, lipid and glucose metabolism, appetite, and inflammation (Zhang et al., 2021). In the current study, serum levels of butyric acid under acute heat stress did not differ significantly among all treatment groups. However, 2-Aminoisobutyric acid levels were up-regulated in all treatment groups. Relevant studies suggested that cold stress directly increases butyric acid production, which responds to cold conditions by boosting adipose thermogenesis (Li et al., 2019; Wang et al., 2020). Butyrate in intestinal contents plays an important anti-inflammatory role in regulating the interaction between fat cells and macrophages by reducing the decomposition of fat and inhibiting inflammatory signaling pathways (Ohira et al., 2013; Mollica et al., 2017). For example, in some inflammatory bowel diseases, levels of SCFAs (such as acetate, propionate, and butyrate) in intestinal contents are reduced (Parada et al., 2019). In our study, it was also found that the content of SCFAs in serum metabolites was decreased, indicating that the increase of intestinal permeability and intestinal epithelial cell damage in rainbow trout under acute heat stress may be related to the changes in SCFAs levels. The effect of butyrate on intestinal barrier function is mediated by TJPs, whose assembly is associated with amp protein kinase (AMPK) activation (Zhang et al., 2006; Zheng and Cantley, 2007). Peng et al. (2009) found that butyrate enhances the intestinal barrier by regulating the assembly of tight junctions. In our results, butyrate content was decreased in the heat stress group, indicating that the down-regulation of intestinal TJPs-related gene expression was related to the decrease of serum butyrate content in rainbow trout under heat stress. These results suggest that the enhancement of intestinal barrier function by increasing the level of gut SCFAs plays an important role in improving the anti-stress ability of rainbow trout.

Although this study expands our understanding of how intestinal microbiota drives host metabolism under heat stress, there are still some limitations. The causal relationship between intestinal microbiota and host metabolism under chronic stress is an important issue to be considered and further studied. Furthermore, there are limited experimental methods to study the two complex issues of intestinal microbiota and host metabolism. Therefore, more advanced research methods, such as metagenomics, should be considered to compare our results.

## Conclusion

Our results suggest that acute heat stress reduces the relative abundance and diversity of the intestinal microbiota of rainbow trout, while increasing the proportion of Mycoplasma, Firmicutes, and Tenericutes in the intestinal microbiota. The results of non-targeted metabonomics and correlation analysis showed that the changes of metabolites related to amino acids, vitamins, and SCFAs in serum of rainbow trout under acute heat stress were closely correlated with the decrease of relative abundance of various intestinal microbiota, especially *Morganella*, *Enterobacter*, *Lactobacillus*, *Lawsonia*, and *Cloacibacterium*. These results suggest that there is a strong association between the gut microbiome and its host, and that disruption of homeostasis and metabolic dysfunction in the host may be driven by changes in the gut microbiome. In addition, the gut microbiome of acute heat stress rainbow trout could mediate metabolite transfer through the gut barrier by affecting its integrity, including significant changes in gut morphology, permeability, antioxidant capacity, and pro-inflammatory cytokine levels.

## Data Availability Statement

The original contributions presented in the study are publicly available. This data can be found here: http://doi.org/10.6084/m9.figshare.19329821.

## Ethics Statement

The animal study was reviewed and approved by the Ethics Committee of School of Life Sciences, Lanzhou University. Written informed consent was obtained from the owners for the participation of their animals in this study.

## Author Contributions

CZ implemented the study and wrote the manuscript. SY, WK, PG, and YL assisted in conducting the experiment and collecting samples. RL and JW designed the experiment. JW revised the manuscript. All authors contributed to the study and supported the submitted version.

## Conflict of Interest

The authors declare that the research was conducted in the absence of any commercial or financial relationships that could be construed as a potential conflict of interest.

## Publisher’s Note

All claims expressed in this article are solely those of the authors and do not necessarily represent those of their affiliated organizations, or those of the publisher, the editors and the reviewers. Any product that may be evaluated in this article, or claim that may be made by its manufacturer, is not guaranteed or endorsed by the publisher.

## Footnotes

1
www.biocloud.net

2
http://www.arb-silva.de

3
https://qiime2.org/

4
http://huttenhower.sph.harvard.edu/lefse/

5
http://cytoscape.org/
