# Supplementary material for: Association of Gut Microbiota With Metabolism in Rainbow Trout Under Acute Heat Stress
Source: Front Microbiol. 2022 Mar 30;13:846336. doi: 10.3389/fmicb.2022.846336 (PMC9007319; doi:10.3389/fmicb.2022.846336)
Supplement: Supplementary file 1 [file Data_Sheet_1.docx]

Supplementary Material

# Supplementary Figures and Tables

## Supplementary Tables

**Supplementary Table 1. Primers sequence of QRT-PCR**

| Gene | Accession number | Prime sequence (5′-3′) | Product size (bp) |
| --- | --- | --- | --- |
| *Claudin* | KF445436 | F: GGCACGTCTGAGAAACAACA | 245 |
|  |  | R: TAGGAAGTGGCAGCCTGACT |  |
| *Occludin* | GQ476574 | F: CAGCCCAGTTCCTCCAGTA | 341 |
|  |  | R: GCTCATCCAGCTCTCTGTCC |  |
| *ZO-1* | HQ656020 | F: AAGGAAGGTCTGGAGGAAGG | 291 |
|  |  | R: CAGCTTGCCGTTGTAGAGG |  |
| *IL-1β* | AJ223954 | F:ACCGAGTTCAAGGACAAGGA | 181 |
|  |  | R: CATTCATCAGGACCCAGCAC |  |
| *IL-6* | DQ866150 | F:CAATCAACCCTACTCCCCTCT | 91 |
|  |  | R: CCTCCACTACCTCAGCAACC |  |
| *TNF-α* | AJ401377 | F:GGGGACAAACTGTGGACTGA | 208 |
|  |  | R: GAAGTTCTTGCCCTGCTCTG |  |
| *β-actin* | AB196465 | F: TGGGGCAGTATGGCTTGTATG | 165 |
|  |  | R: CTCTGGCACCCTAATCACCTCT |  |

**Supplementary Table 2. Sample sequencing results statistics**

| Sample | Raw Reads | Clean Reads | Effective Reads | AvgLen (bp) | GC (%) | Q20 (%) | Q30 (%) | Effective (%) |
| --- | --- | --- | --- | --- | --- | --- | --- | --- |
| 16 ℃-a | 80443 | 80106 | 76208 | 420 | 53.76 | 99.08 | 96.24 | 94.74 |
| 16 ℃-b | 80038 | 79732 | 75813 | 419 | 53.74 | 99.09 | 96.25 | 94.72 |
| 16 ℃-c | 79829 | 79522 | 75731 | 420 | 53.71 | 99.08 | 96.22 | 94.87 |
| 22.5 ℃-a | 79870 | 79554 | 76653 | 422 | 50.81 | 99.06 | 96.07 | 95.97 |
| 22.5 ℃-b | 79973 | 79658 | 76631 | 421 | 51.87 | 99.06 | 96.14 | 95.82 |
| 22.5 ℃-c | 80109 | 79790 | 78863 | 425 | 47.93 | 99.03 | 95.92 | 98.44 |
| 23.5 ℃-a | 80026 | 79663 | 75866 | 419 | 53.45 | 99.07 | 96.21 | 94.8 |
| 23.5 ℃-b | 79836 | 79506 | 77394 | 419 | 50.92 | 99.1 | 96.24 | 96.94 |
| 23.5 ℃-c | 80152 | 79836 | 78542 | 419 | 51.3 | 99.12 | 96.29 | 97.99 |
| 24.5 ℃-a | 79885 | 79562 | 78101 | 418 | 51.76 | 99.12 | 96.29 | 97.77 |
| 24.5 ℃-b | 79900 | 79596 | 77887 | 412 | 50.84 | 99.17 | 96.45 | 97.48 |
| 24.5 ℃-c | 79856 | 79564 | 77807 | 421 | 52.7 | 99.1 | 96.25 | 97.43 |

**Supplementary Table 3. The proportion of rainbow trout intestinal flora at phylum level**

| Phylum | 16 ℃ | 22.5 ℃ |  | 23.5 ℃ | 24.5 ℃ |
| --- | --- | --- | --- | --- | --- |
| Proteobacteria | 0.319695 | 0.216405 |  | 0.269171 | 0.383035 |
| Firmicutes | 0.289809 | 0.163888 |  | 0.368196 | 0.120018 |
| Tenericutes | 0.001439 | 0.427495 |  | 0.10361 | 0.022913 |
| Fusobacteria | 0.014215 | 0.080396 |  | 0.027233 | 0.421925 |
| Bacteroidetes | 0.138428 | 0.04668 |  | 0.123826 | 0.021098 |
| Actinobacteria | 0.069693 | 0.024033 |  | 0.050148 | 0.009708 |
| Cyanobacteria | 0.04381 | 0.011965 |  | 0.015511 | 0.00523 |
| Spirochaetes | 0.031427 | 0.00726 |  | 0.010035 | 0.00385 |
| Acidobacteria | 0.027408 | 0.007109 |  | 0.011247 | 0.004134 |
| Verrucomicrobia | 0.021579 | 0.005885 |  | 0.007491 | 0.003286 |
| Others | 0.042497 | 0.008884 |  | 0.013534 | 0.004803 |

**Supplementary Table 4. The proportion of rainbow trout intestinal flora at genus level**

| Genus | 16 ℃ | 22.5 ℃ | 23.5 ℃ | 24.5 ℃ |
| --- | --- | --- | --- | --- |
| Mycoplasma | 0.001085 | 0.427271 | 0.103096 | 0.022853 |
| Cetobacterium | 0.008466 | 0.078251 | 0.023608 | 0.38787 |
| Aeromonas | 0.041931 | 0.079806 | 0.061157 | 0.168213 |
| Shewanella | 0.001998 | 0.064735 | 0.094766 | 0.169272 |
| Clostridium_sensu_stricto_1 | 0.005863 | 0.042716 | 0.150486 | 0.044226 |
| Bacteroides | 0.04812 | 0.017811 | 0.080549 | 0.006376 |
| Enterobacter | 0.059338 | 0.017119 | 0.014896 | 0.007384 |
| Lactobacillus | 0.037262 | 0.012662 | 0.023201 | 0.005816 |
| Lawsonia | 0.0385 | 0.008191 | 0.012434 | 0.004405 |
| Romboutsia | 0.001861 | 0.028801 | 0.007811 | 0.021666 |
| Others | 0.755576 | 0.222636 | 0.427996 | 0.16192 |

## Supplementary Figures


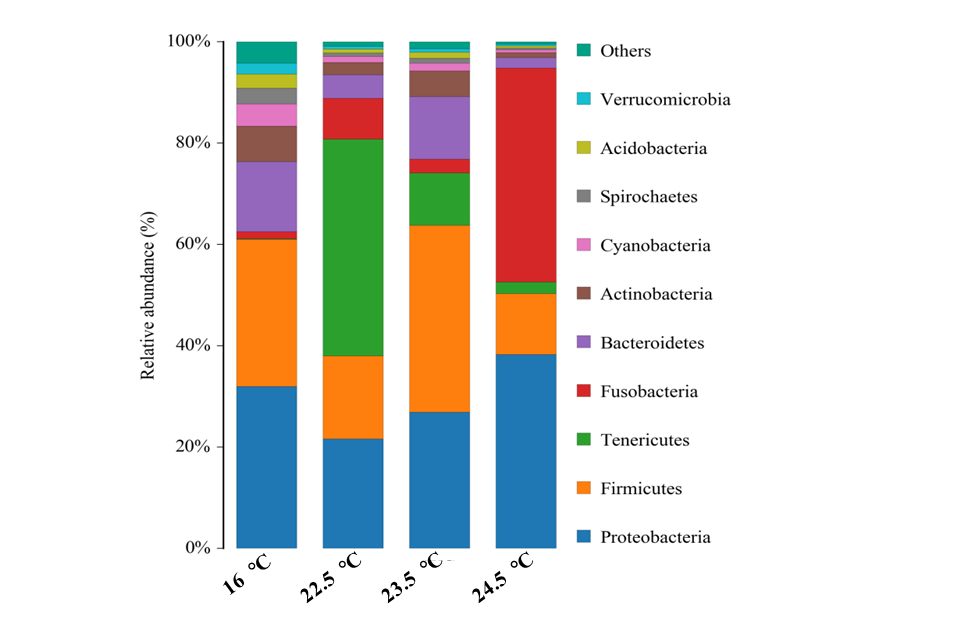


**Supplementary Figure 1.** Histogram of intestinal microflora differences at phylum level in different stress temperature groups.


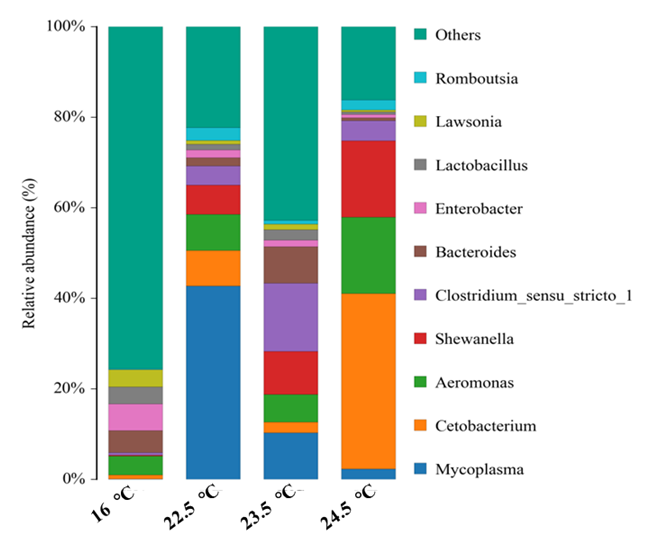


**Supplementary Figure 2.** Histogram of intestinal microflora differences at genus level in different stress temperature groups.


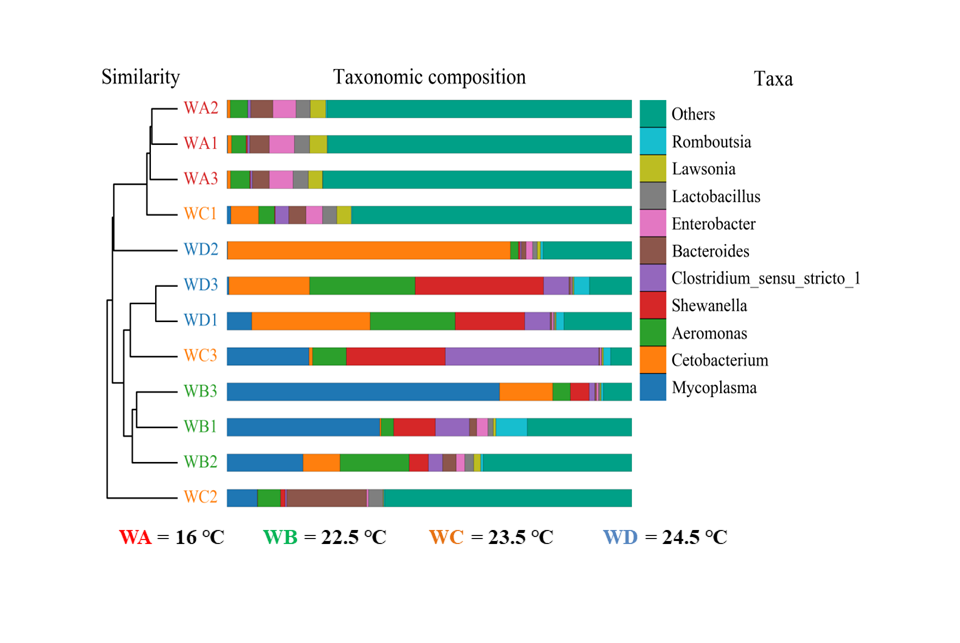


**Supplementary Figure 3.** Analysis of the difference of intestinal microflora in different stress temperature groups.


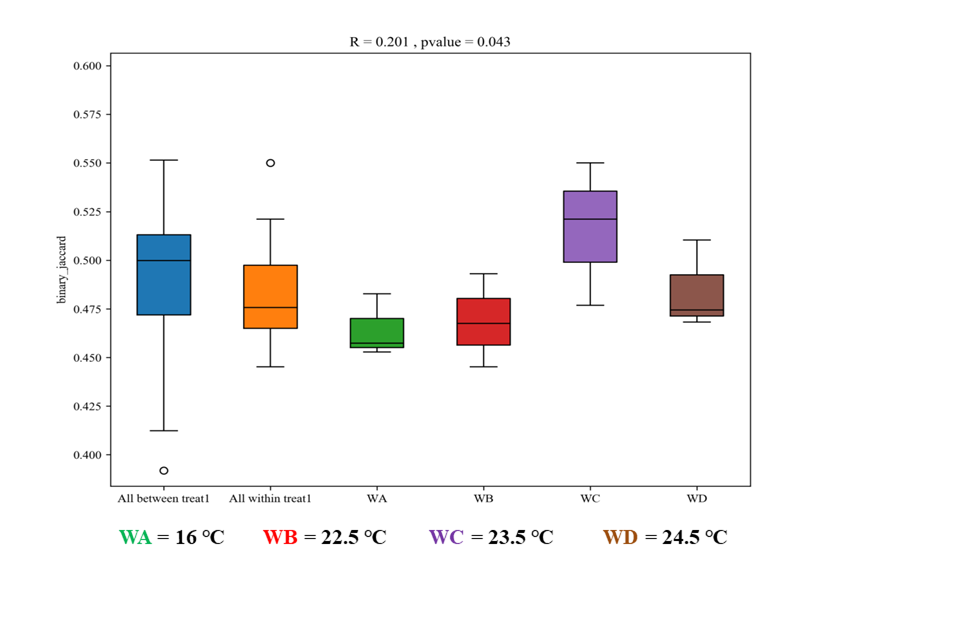


**Supplementary Figure 4.** Analysis of the difference of intestinal microflora in different stress temperature groups. WA, 16 °C control group; WB, 22.5 °C stress group; WC, 23.5 °C stress group; WD, 24.5 °C stress group.


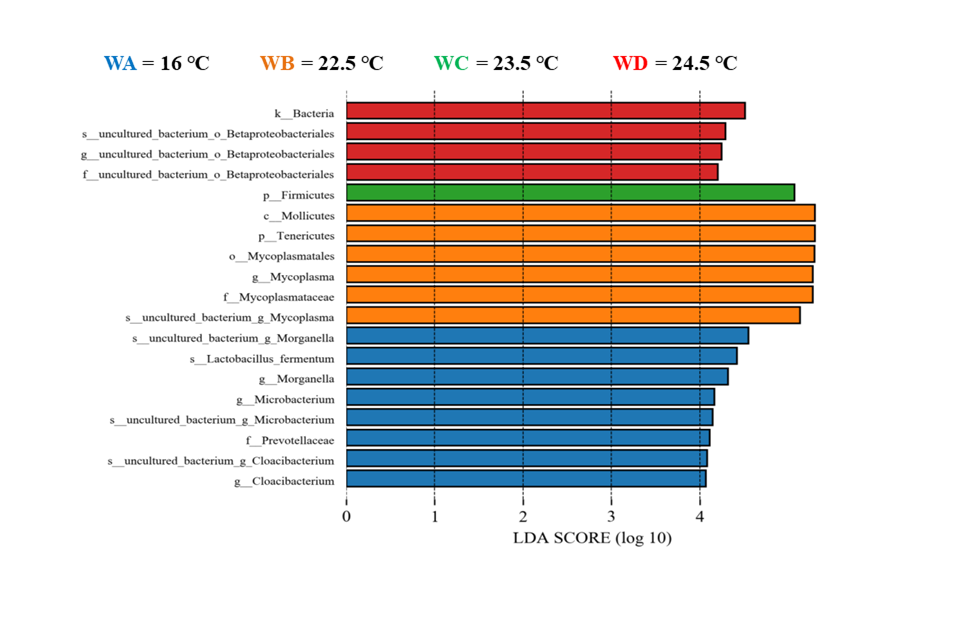


**Supplementary Figure 5.** Analysis of the difference of intestinal microflora in different stress temperature groups.


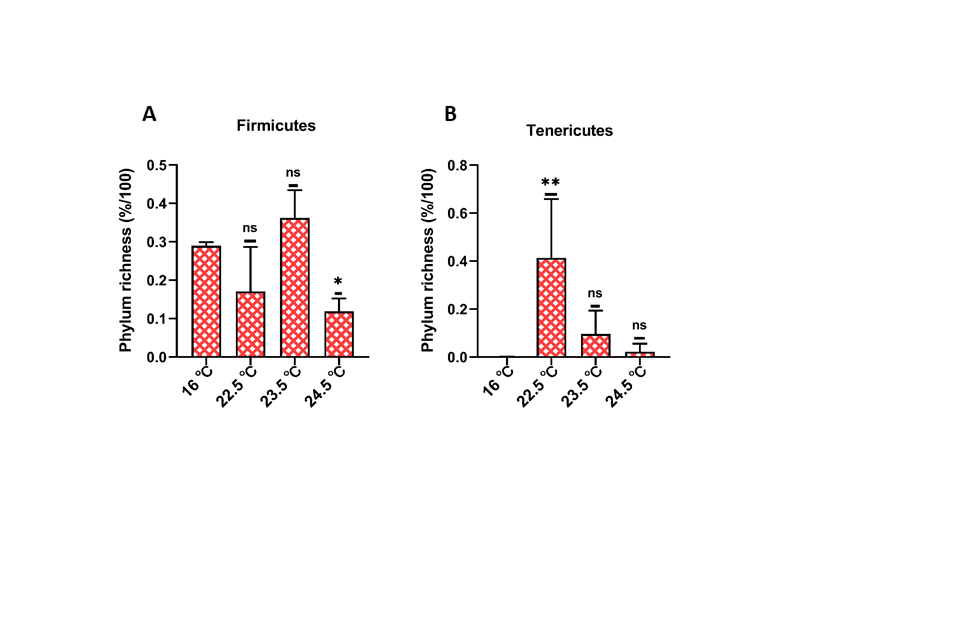


**Supplementary Figure 6.** Biomarkers of discriminative bacteria (Phylum) in different temperature groups identified. *P < 0.05; **P < 0.01; ns, no significance.
